# 25-Hydroxyvitamin D as a biomarker of vitamin D status in plaque psoriasis and other dermatological diseases: a cross-sectional study

**DOI:** 10.1590/1516-3180.2022.0164.R1.19052022

**Published:** 2022-08-12

**Authors:** Shirley Braga Lima Gamonal, Aloisio Carlos Couri Gamonal, Nathália Couri Vieira Marques, Marcos Antônio Fernandes Brandão, Nádia Rezende Barbosa Raposo

**Affiliations:** I MD, MSc, PhD. Researcher, Physician and Professor, Núcleo de Pesquisa em Dermatologia (NUPEDE), Faculty of Medicine, Universidade Federal de Juiz de Fora (UFJF), Juiz de Fora (MG), Brazil; and Researcher, Núcleo de Pesquisa e Inovação em Ciências da Saúde (NUPICS), Faculty of Pharmacy, Universidade Federal de Juiz de Fora (UFJF), Juiz de Fora (MG), Brazil.; II MD, MSc, PhD. Physician and Professor, Núcleo de Pesquisa em Dermatologia (NUPEDE), Faculty of Medicine, Universidade Federal de Juiz de Fora (UFJF), Juiz de Fora (MG), Brazil.; III Undergraduate Student, Medicine, Núcleo de Pesquisa em Dermatologia (NUPEDE), Faculty of Medicine, Universidade Federal de Juiz de Fora (UFJF), Juiz de Fora (MG), Brazil.; IV PhD. Pharmacist and Professor, Núcleo de Pesquisa e Inovação em Ciências da Saúde (NUPICS), Faculty of Pharmacy, Universidade Federal de Juiz de Fora (UFJF), Juiz de Fora (MG), Brazil.; V MSc, PhD. Pharmacist and Professor, Núcleo de Pesquisa e Inovação em Ciências da Saúde (NUPICS), Faculty of Pharmacy, Universidade Federal de Juiz de Fora (UFJF), Juiz de Fora (MG), Brazil.

**Keywords:** Psoriasis, Vitamin D, Retrospective studies, Brazil, Prevalence, Plaque psoriasis, 25-Hydroxyvitamin D, Seasonal variation, Fitzpatrick phototypes, Retrospective cross-sectional study

## Abstract

**BACKGROUND:**

Hypovitaminosis D is a public health problem associated with several chronic inflammatory and immunological diseases, including psoriasis.

**OBJECTIVES:**

This study aimed to determine the prevalence of hypovitaminosis D in patients with plaque psoriasis. A comparison was made between vitamin D levels in patients with psoriasis and those with other non-inflammatory dermatoses without photosensitivity. In addition, it evaluated the effects of the patients’ Fitzpatrick skin phototype and the season of the year on the serum levels of vitamin D.

**DESIGN AND SETTINGS:**

A retrospective cross-sectional study was conducted at an outpatient clinic in a university center in Juiz de Fora (MG), Brazil.

**METHODS:**

A review of dermatology patients’ demographic data, including skin phototype and serum levels of 25-hydroxyvitamin D [25(OH)D], over 12 months in 2016.

**RESULTS:**

This study included 554 patients: 300 patients allocated to the plaque psoriasis group and 254 control patients with other dermatological diseases. Regarding the season of the year, 229, 132, 62, and 131 participants were evaluated in summer, autumn, winter, and spring, respectively. As for the skin phototype, 397, 139, and 18 patients had phototypes III, IV, and V, respectively. The serum levels of 25(OH)D were significantly lower in the psoriasis group (24.91 ± 7.16 ng/mL) than in the control group (30.37 ± 8.14 ng/mL).

**CONCLUSIONS:**

Hypovitaminosis D (< 30 ng/mL) was present in 76.66% of patients with psoriasis versus 53.94% of control patients. Vitamin D deficiency (< 20 ng/mL) was observed in 25% of the patients with psoriasis versus 8.66% in the control group (P < 0.001). The season and patient’s skin phototype were independent predictors of serum vitamin D levels.

## INTRODUCTION

Psoriasis is a chronic inflammatory disease with a genetic predisposition involving the skin, joints, and immunological effector mechanisms, affecting approximately 2–5% of the world population. The pathogenesis is multifactorial, involving innate and adaptive immunity, and potentially associated with several comorbidities.^1,2^

Vitamin D is a steroid hormone that acts genomically and non-genomically in different metabolic processes in most tissues. In the skin it plays several important biological functions in the physiology of keratinocytes and cells of innate and adaptive immunity. Several studies have demonstrated a high prevalence of vitamin D deficiency in the general population and its various associations with bone, autoimmune, inflammatory, hormonal, cardiac, and neoplastic diseases.^3,4,5,6,7,8^ Scientific literature suggests an association between psoriasis and inadequate levels of vitamin D.^9,10^ Therefore, it is believed that the prevalence of hypovitaminosis D is higher in patients with psoriasis. Although vitamin D analogs treat psoriasis, its exact mechanism of action and relationship with the disease is unclear.^11^

## OBJECTIVES

The present study aimed to evaluate the serum levels of 25-hydroxyvitamin D [25(OH)D] in patients with plaque psoriasis. In addition, a comparison was made with levels in dermatology patients with other non-inflammatory dermatoses without photosensitivity. This study also aimed to explore the factors associated with vitamin D synthesis. It evaluated the effect of the patient’s Fitzpatrick skin phototype^12^ and season of the year on the serum levels of 25(OH)D.

## METHODS

### Study design and ethics statement

A cross-sectional, retrospective, and comparative study was conducted from January to December 2016 at the Dermatology Service of the University Hospital of the Faculty of Medicine of Universidade Federal de Juiz de Fora (UFJF). The study included 554 patients: 300 patients with plaque psoriasis and 254 control patients with other dermatological diseases. The study reviewed 650 medical records: 350 patients treated at the psoriasis outpatient clinic with a standardized medical record, including serum levels of 25(OH)D, and 300 patients treated at the general dermatology outpatient clinic. Exclusion of patients in both groups was based on the lack of accordance with the inclusion and exclusion criteria, missing data in the medical records, and the patient belonging to a different geographic region. Fifty patients were excluded from the psoriasis group and 46 from the general dermatology group. As the medical records for this study were obtained from the Dermatology Service of a university hospital, the data were collected by postgraduate doctors and supervised by doctors, and standardized medical records were used. To minimize geographic effects on vitamin D levels, all included patients were from Minas Gerais State, Zona da Mata region, southeastern Brazil. The inclusion criteria were patients of both sexes, aged between 18 and 60 years, with a clinical or histopathological diagnosis of plaque psoriasis. The exclusion criteria were patients with other clinical forms of psoriasis; severe and decompensated systemic diseases (hepatic, renal, metabolic, and cardiac); thyroid and parathyroid diseases; malignant neoplasms, AIDS, and pregnancy; oral supplementation of vitamin D, bisphosphonates, systemic corticosteroids, and calcium; treatment with phototherapy or use of sunscreens; use of topical vitamin D analogs such as calcipotriol; and diseases with altered intestinal absorption or other autoimmune and photosensitivity diseases.

This retrospective study was performed after the research was approved by our institution’s ethics committee (protocol 3.142.153; approved on November 2, 2019, by the Research Ethics Committee of the University Hospital, UFJF). All the procedures involved in this study were in accordance with the Declaration of Helsinki of 1975, updated in 2013.

### Clinical predictors and laboratory screening

The standardized medical records of each patient were reviewed, and the following variables were evaluated: sex, age, Fitzpatrick skin phototype, and season of the year in which serum levels of 25(OH) vitamin D were measured. All serum sample vitamin D levels were analyzed at the Biochemistry Laboratory of the University Hospital using the chemiluminescence technique (ARCHITECT 25-OH Vitamin D, Abbott Diagnostics, Lake Forest, Illinois, United States). According to the American Association of Endocrinology,^13^ the following parameters were adopted: values lower than 20 ng/mL were considered deficient, values from 20 ng/mL to lower than 30 ng/mL were considered insufficient, and values equal to or above 30 ng/mL were considered sufficient.

### Statistical analysis

A descriptive data analysis was performed, and the normality of distribution was assessed using the Shapiro–Wilk test. Levene’s test was used to test for homogeneity of variance. When the distribution and homogeneity of variance were met, Student’s t-test was used to test the differences in quantitative variables between the two groups. The Chi-square test (χ2) or Fisher’s exact test for less than five data points was used to test possible differences in the proportions of qualitative variables. Multivariate linear regression analysis was used to determine independent predictors of serum vitamin D. vitamin D was used continuously in multivariate regression. Season, skin phototype, sex, and age were used as determining variables. The significance level was set for all statistical analyses at 5% (P < 0.05). Analyses were performed using the R software package for Windows [R Core Team (2019); version 3.4.4 (R Foundation for Statistical Computing, Vienna, Austria) and URL HTTPS://WWW.R-PROJECT.ORG/].

## RESULTS

The demographic characteristics and parameters of the two groups are shown in Table 1. Our sample consisted of 554 patients, 300 with plaque psoriasis (54.15%) and 254 patients (45.85%) with other dermatoses.

**Table 1.** Demographic characteristics and serum 25(OH)D concentration in patients with plaque psoriasis and controls

Variables	Psoriasis n (%) 300 (54.15)	Controls n (%) 254 (45.85)	P value
**Age in years (mean ± SD)**	47.23 ± 12.82	41.59 ± 12.09	0.000*
**Male/Female (n)**	161/139	55/199	0.000*
**Prevalence in men (%)**	53.6	21.6	0.000*
**Fitzpatrick skin phototype, [n (%)]**			NA
III	178 (59.33)	219 (73.0)	
IV	105 (35.0)	34 (11.33)	
V	17 (5.66)	1 (0.33)	
**Season of the year during test, [n (%)]**			NA
Autumn	56 (42.9)	76 (57.6)	
Winter	23 (37.1)	39 (62.9)	
Spring	57 (43.5)	74 (56.5)	
Summer	164 (71.6)	65 (28.4)	
**25(OH)D**			
[(Mean ± SD), (ng/mL)]	24.91 ± 7.16	30.37 ± 8.14	0.000*
Minimum	9.17	13.20	
Maximum	48.0	57.0	
**< 20 ng/mL, [n (%)]**	75 (25.00)	22 (8.66)	0.000*
**Between 20 and 30 ng/mL, [n (%)]**	155 (51.66)	11 5(45.28)	0.132
≥ 30 ng/mL, [n (%)]	70 (23.34)	117 (46.06)	0.000*

25(OH)D = 25-hydroxyvitamin D; NA = not applicable; SD = standard deviation; *P < 0.0001.

**Figure 1.**
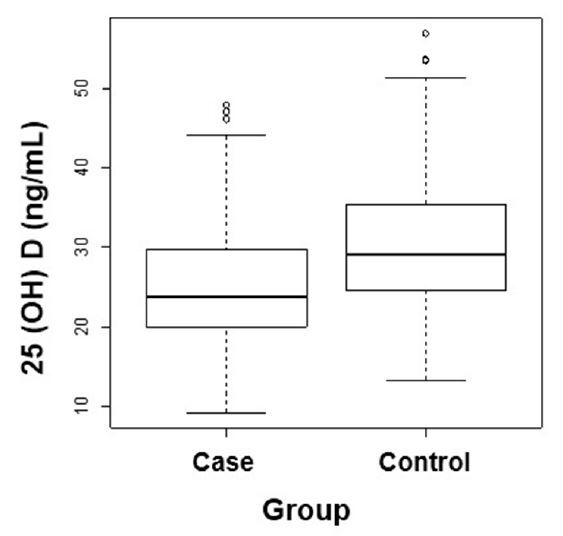
Comparison of serum 25-hydroxyvitamin D [25(OH)D] concentration between the psoriasis and control groups.

The mean age in the case group was significantly higher (47.23 ± 12.82 versus 41.59 ± 12.09 years; P < 0.001). Regarding sex distribution, 338 were women, and 216 were men. The distribution by sex showed statistically significant differences between the two groups (P < 0.001). The case group had more men (53.6%), and the control group had more women (78.4%). Serum 25(OH)D levels were significantly lower in the psoriasis group (24.91 ± 7.16 ng/mL) than in controls (30.37 ± 8.14 ng/mL), with P < 0.001 **(** Figure 1 **).**

The serum 25(OH)D concentration was significantly lower in patients with psoriasis than in control subjects (P < 0.001).

**Figure 2.**
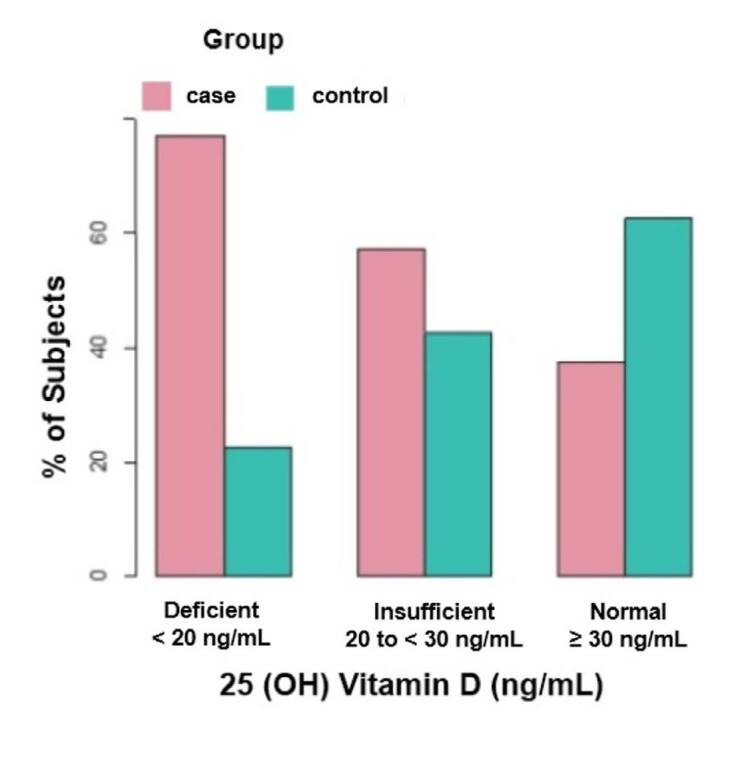
Percentage of patients according to the serum level of 25-hydroxyvitamin D [25(OH)D].

Regarding the skin phototype, there was a predominance of phototype III (397 patients, 71.66%), followed by phototype IV (139 patients, 25.09%), and 18 patients with phototype V (3.25%). Serum levels of 25(OH)D were assessed more frequently during the summer (229 patients, 41.34%) and to a lesser extent during the winter (62 patients, 11.19%). Patients with psoriasis had vitamin D deficiency levels (< 20 ng/mL) in 25% of cases versus 8.66% in controls (P < 0.001). The levels of insufficiency (between 20 and 30 ng/mL) were 51.66% in patients with psoriasis versus 45.28% in controls. In contrast, levels ≥ 30 ng/mL were present in 46.06% of the control group and 23.34% of the case group (P < 0.001). Therefore, hypovitaminosis D (< 30 ng/mL) was observed in 76.66% of patients with psoriasis versus 53.94% of control patients **(** Figure 2 **)**.

Deficient (< 20 ng/mL), insufficient (between 20 and 30 ng/mL), or normal (≥ 30 ng/mL).

The results of the multivariate linear regression, in which the determinant variables of serum vitamin D levels were studied, are shown in Table 2 (adjusted model R^2^= 0.21, P < 0.001). There was a positive association between the summer season and vitamin D serum levels (β coefficient: 6.64, confidence interval [CI]: 4.56 to 8.72). Regarding skin phototypes, there was an inverse association between vitamin D levels and the highest phototype classifications, such as phototype V (β coefficient: -5.06 CI: -8.54 to -1.59). There were no significant effects of age or sex.

**Table 2.** Multivariate linear regression model - analysis of independent predictors of serum 25(OH)D levels in the cohort

Predictors	β coefficient	P value	95% CI
**Group**			
Psoriasis^*^			
Control	6.94	0.000^€^	5.53 – 8.34
**Sex**			
Female^*^			
Male	1.24	0.06	−0.08 – 2.58
**Age**	−0.02	0.22	−0.07 – 0.01
**Season**			
Winter^*^			
Autumn	2.98	0.007	0.81 – 5.15
Spring	1.66	0.13	−0.50 – 3.83
Summer	6.64	0.000^€^	4.56 – 8.72
**Skin phototype**			
III^*^			
IV	0.45	0.54	−1.00 – 1.95
V	-5.06	0.000^€^	−8.54 – −1.59

* Reference Category ^€^P < 0.001 Adjusted model *R* ^2^= 0.21 P < 0.001; CI = confidence interval.

## DISCUSSION

This study involved 554 patients, with 300 patients allocated to the plaque psoriasis group and 254 control patients with other dermatological diseases. Regarding the season of the year, 229, 132, 62, and 131 participants were evaluated in summer, autumn, winter, and spring, respectively. For the skin phototype, 397, 139, and 18 patients had phototypes III, IV, and V, respectively. The serum levels of 25(OH)D were significantly lower in the psoriasis group (24.91 ± 7.16 ng/mL) than in the control group (30.37 ± 8.14 ng/mL). A negative association was found among 25(OH)D, psoriasis, and phototypes IV and V, and a positive association between 25(OH)D and summer.

The importance of vitamin D in systemic homeostasis has attracted great interest in the scientific community, with numerous studies on its physiology and impact on global health. However, several issues remain controversial, such as the reasons for a substantial portion of the world population having low levels of vitamin D, the best laboratory test for vitamin D dosage, and the parameters to be used to properly define the cut-off (point) to express vitamin D sufficiency, insufficiency, or deficiency.^13,14^

According to the Central and Eastern European Expert Consensus Statement,^15^ recently published serum creatinine measurements have been advised in individuals with a 25(OH)D concentration of < 10 ng/mL or > 100 ng/mL. As only one patient in the study presented with levels of vitamin D < 10 ng/mL, the creatinine levels in the patients were not evaluated, and the creatinine level of this single patient was normal.

According to the American Academy of Endocrinology guidelines,^13^ a 66.24% prevalence of hypovitaminosis D (< 30 ng/mL) was found in the cohort. In Brazil, studies have shown a prevalence of hypovitaminosis D in adults of approximately 33 to 71.2%.^14,16,17,18,19^ Moreover, the mean was significantly lower in the psoriasis group (24.91 ± 7.16 versus 30.37 ± 8.14 ng/mL; P < 0.001). Approximately 25% of the patients with psoriasis were deficient (< 20 ng/mL). The prevalence of hypovitaminosis was 76.66% in patients with psoriasis versus 53.94% in the control group. Levels considered adequate (≥ 30 ng/mL) were present in 46.06% of the control group and 23.34% of the case group.

Our data coincided with those of Orgaz-molina et al.,^9^ who found that 25.6% of patients with psoriasis were deficient, and Ricceri et al.,^10^ who found a 68% rate of deficiency and 97% rate of insufficiency in patients with psoriasis versus 10% and 53% in the control group, respectively. The prevalence levels of hypovitaminosis were similar to those in the Brazilian study by Zuchi et al.,^20^ who analyzed 40 patients: 20 with psoriasis (15 with mild plaque form and 5 palmoplantar) and 20 control patients. This study was conducted from July to September 2013 in Curitiba City, South Brazil, and the most frequent skin phototypes were I and II. In the analysis of 25(OH)D, a prevalence of 85% for hypovitaminosis D (< 30 ng/mL) was found in the studied sample. When analyzing the levels of deficiency in patients with psoriasis, the results were 25% versus 20% in the controls. However, no statistically significant differences were observed between the studied populations.

Furthermore, several factors can influence the prevalence of deficiency and insufficiency, such as race, ultraviolet radiation index, dietary intake, and year’s season.^21^ In the multivariate analysis, phototype and season of the year were the independent variables statistically significantly associated with 25(OH)D serum concentrations.

Juiz de Fora is a city in the Zona da Mata region of Minas Gerais, located in the intertropical zone. Therefore, it receives a large amount of sunlight throughout the year, and this study was conducted in a city with a high ultraviolet index, ranging from moderate to high. Although more than 40% of the tests were performed during summer and to a lesser extent in winter (11%), a high insufficiency rate of 25(OH)D was detected.

In a review carried out by Corrêa^22^ in Brazil, he concluded that the ultraviolet index (UVI) values observed usually reach the highest UVI scales recommended by the World Health Organization (WHO), with very high (UVI between 8 and 10) or extreme (UVI greater than 11) damage to human health. In the North and Northeast regions of the country, similar values can be observed before 9 am throughout the year. In the south and southeast, at solar noon, UVI values are characterized by marked seasonality, with extreme UVI values in summer and between medium and high in winter. However, it is during the summer in the southeast region of the country where Brazilian records of UVI episodes above 15 are observed. This new knowledge about the distribution of UVI in Brazil is important to consider with respect to the problem of hypovitaminosis D, which may be associated with other factors, such as genetic and nutrigenomic polymorphisms and little sun exposure.^22^

Another factor involved in the serum concentration of vitamin D is the amount of melanin in the skin. A reduction in serum 25(OH)D concentrations in people with higher skin phototype classifications is expected because they synthesize less vitamin D when exposed to the same amount of radiation compared to a person with a lower skin phototype classification. This is explained by the fact that melanin competes for the photon of ultraviolet B radiation (UVB), which promotes the photolysis of 7-dehydrocholesterol and triggers the synthesis of vitamin D in the skin. Consequently, radiation exposure should be longer in higher skin phototype classifications.^23^ More than 70% of the patients in our sample had skin phototype III, and the prevalence of hypovitaminosis D was still high. The literature data indicates disagreement about the phototype variable, such as those by Glass et al.,^24^ who studied the relationship between vitamin D, skin pigmentation, and exposure to UV rays in the United Kingdom. The study analyzed 1400 white women. It was observed that individuals with the highest phototype classifications (III and IV) had higher serum levels of 25(OH)D (mean 32.9 ng/mL) when compared to those with low phototype classifications (types I and II) (mean 28.5 ng/ml, P < 0.0001). The data showed an inclination towards sun-seeking behavior in darker-skinned patients, which correlated positively with vitamin D status. Malvy et al.^25^ conducted a similar study in France involving 1191 individuals and found that serum 25(OH)D levels were lower in fair-skinned individuals (P < 0.024).

The exacerbation of psoriasis in winter may be partly due to low sun exposure and the subsequent low vitamin D production in the skin. Therefore, the therapeutic effect of UVB therapy in treating psoriasis may be, at least in part, mediated by UVB causing the synthesis of vitamin D in the skin. In addition, UVB therapy increased serum 25(OH)D levels in patients with psoriasis in parallel with disease improvement.^26^ The exposure of the skin to sunlight is the major source of vitamin D. Moreover, the epidermis and hair follicle keratinocytes express the hydroxylases needed to produce the active hormone 1,25-Dihydroxyvitamin D [1,25(OH)2D], and vitamin D receptor has been shown on keratinocytes.

Vitamin D appears to influence the innate and acquired immune systems with complex effects, which are still not completely elucidated. It has been shown that 1 α-hydroxylation produces active hormones within different immune system cells, where it exerts autocrine and paracrine effects. In contrast, vitamin D mainly inhibits the acquired immune system by reducing the expression of MHC class II and co-signaling molecules on antigen-presenting cells, decreasing the activity of TH1 and TH17 cells, and upregulating regulatory T cells. The final result is the promotion of regulatory and protective phenotypes of T-cells.^27^

The benefits of vitamin D analogs for psoriasis treatment are well established. A topical vitamin D analog is the first-line choice for managing psoriasis, either alone or in combination with topical corticosteroids. Unlike corticosteroids, which can be associated with tachyphylaxis, topically administered vitamin D analog treatment is long-term and effective without side effects in patients of all ages.^28^

Stanescu et al.,^29^ in their review of the systemic use of vitamin D in psoriasis, examined the pros and cons of this treatment to determine whether systemic vitamin D would be a feasible therapeutic option for these patients and whether more large-scale studies are needed to determine the efficacy, optimal dosing, and adverse effects of vitamin D administration in patients with psoriasis. Genetic variation in vitamin D metabolism can lead to a personalized vitamin D response. Moreover, biomarkers of vitamin D status different from 25(OH)D status have been identified in new metabolic pathways of vitamin D.^30^

Although sunlight was the primary source of vitamin D during more than 99% of human evolution, it is clear that mainly owing to increased longevity, people need to try to accomplish a delicate balance between limiting sunlight exposure, avoiding skin damage, and optimizing vitamin D status. In many cases, this balance implies that vitamin D supplementation is necessary.

A Brazilian study conducted by Coutinho et al.^31^ in 174 fishermen analyzed the relationship between sun exposure index, vitamin D levels, and clinical changes in the skin caused by the sun. Vitamin D deficiency was verified in only 11.46% of the patients due to chronic sun exposure in Brazil’s northeast region, with high levels of UVI throughout the year. The lack of association between our study and that conducted by Coutinho et al. can be explained by the fact that our study showed a higher prevalence of vitamin D deficiency, as it was performed in a geographic region with a variation in sun exposure according to the season of the year, as well as the presence of atmospheric pollution in the Southeast region. On the other hand, our findings are similar to those of Cabral et al.,^32^ found in another Brazilian study in the Northeast region.

To the best of our knowledge, this is the first Brazilian study to assess the prevalence of hypovitaminosis D in dermatological patients for 12 months in an expressive cohort. Therefore, the data from this study can be considered representative of a considerable proportion of dermatological patients, including patients with psoriasis in Brazil.

The limitations of our study include the absence of a dietary and sun-exposure survey (with time and duration of exposure). In addition, 25(OH)D production and degradation is a continuous process. Therefore, establishing an ideal period to study the effects of UV radiation on vitamin D production and its action on immunosuppression is a challenge in clinical research. Consequently, it needs to be better evaluated in prospective studies. In addition, as this study was cross-sectional, the patients were not followed up over a long period of restrictive selection criteria.

## CONCLUSION

Considering the geographic location in which the study was carried out, with moderate to high levels of ultraviolet radiation throughout the year and the predominance of skin phototype III, it can be concluded that daily solar radiation was insufficient to promote the adequate synthesis of 25(OH)D. Furthermore, vitamin D deficiency was greater in the psoriasis group. A negative association was found among 25(OH)D, psoriasis, and phototypes IV and V, and a positive association between 25(OH)D and summer. Future randomized, blinded, long-term studies investigating the role of vitamin D supplementation in psoriasis are necessary.
